# Did E-Cigarette Users Notice the New European Union’s E-Cigarette Legislation? Findings from the 2015–2017 International Tobacco Control (ITC) Netherlands Survey

**DOI:** 10.3390/ijerph16162917

**Published:** 2019-08-14

**Authors:** Dirk-Jan A. van Mourik, Gera E. Nagelhout, Bas van den Putte, Karin Hummel, Marc C. Willemsen, Hein de Vries

**Affiliations:** 1 Department of Health Promotion, Maastricht University (CAPHRI), Maastricht, 6229 HA Limburg, The Netherlands; 2 Department of Family Medicine, Maastricht University (CAPHRI), Maastricht, 6229 HA Limburg, The Netherlands; 3 IVO Research Institute, The Hague, 2595 AA Zuid Holland, The Netherlands; 4 Department of Communication, University of Amsterdam (ASCoR), Amsterdam, 1001 NG Noord Holland, The Netherlands; 5 Netherlands Expertise Center for Tobacco Control (NET), Trimbos Institute, Utrecht, 3521 VS Utrecht, The Netherlands

**Keywords:** electronic cigarette, health warnings, noticing

## Abstract

This study examined to what extent e-cigarette users noticed the European Union’s new legislation regarding e-cigarettes, and whether this may have influenced perceptions regarding addictiveness and toxicity. Data were obtained from yearly surveys (2015–2017) of the International Tobacco Control (ITC) Netherlands Survey. Descriptive statistics and Generalized Estimating Equations were applied. About a third of the e-cigarette users noticed the text warning (28%) and the leaflet (32%). When compared to tobacco-only smokers, e-cigarette users showed greater increases in perceptions regarding addictiveness (β = 0.457, *p* = 0.045 vs. β = 0.135, *p* < 0.001) and toxicity (β = 0.246, *p* = 0.055 vs. β = 0.071, *p* = 0.010). In conclusion, the new legislation’s noticeability should be increased.

## 1. Introduction

**Figure 1:**
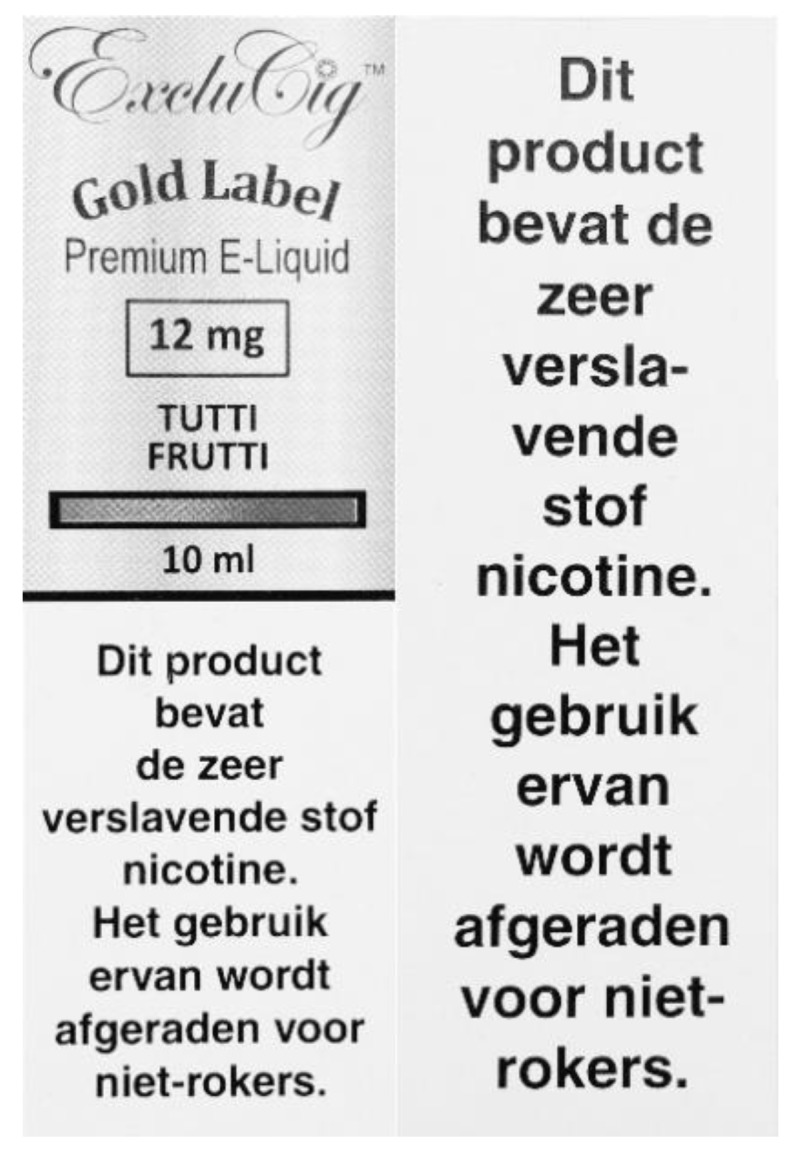
Example of the EU’s new text warning on an e-liquid packet (Left: the front of the e-liquid packet with the warning, “This product contains the highly addictive substance nicotine. Its use is discouraged for non-smokers”; right: the back of the packet with the same warning).

**Figure 2:**
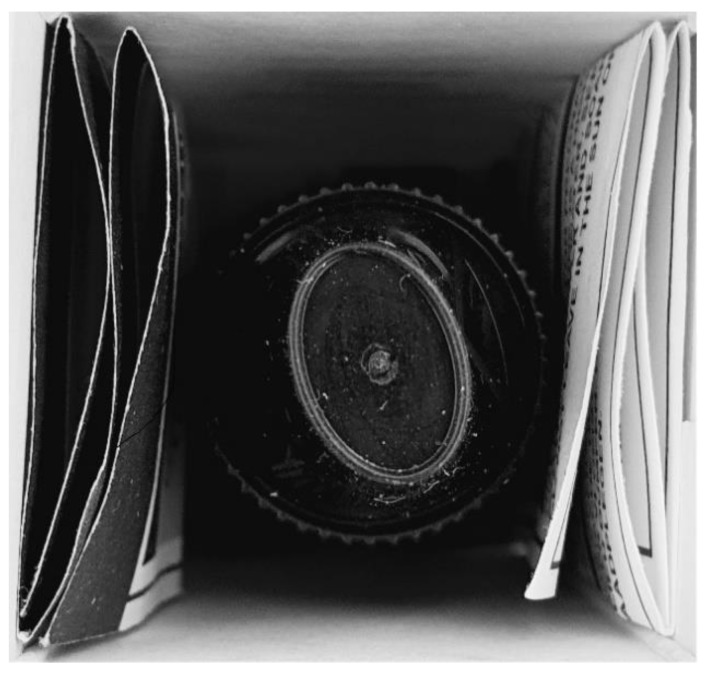
The placing of the EU’s new e-cigarette leaflet inside an e-liquid packet.

**Figure 3:**
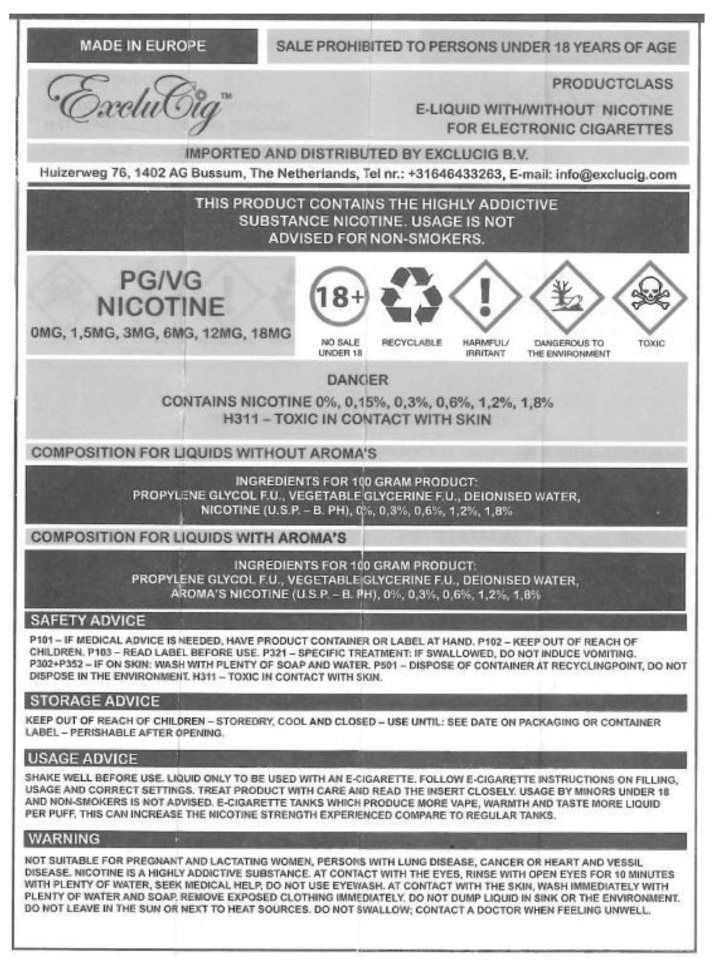
The EU’s new e-cigarette leaflet.

Electronic cigarettes (e-cigarettes) are increasingly used in the Netherlands, with a prevalence of 3.5% in 2016 [1], while smoking prevalence was 22% in 2018 [2], with around 16% of Dutch smokers regularly using e-cigarettes (dual-users) [3]. Although e-cigarettes may have better short- and midterm health-risk profiles than regular cigarettes [4], they contain toxins [5], their long-term safety profile is unknown [4], and they are addictive when containing nicotine. It has been argued that e-cigarette users should be well-informed about this [6]. As of May 2016, in the European Union (EU), the new Tobacco Products Directive required that both the unit and outside packet of e-cigarette products with nicotine must include a text warning on at least 30% of the two largest surfaces, stating that nicotine is addictive (Figure 1). All e-cigarette packets now also have to include a leaflet with information about, amongst other things, the addictiveness and toxicity of e-cigarettes (Figure 2 and Figure 3). This new legislation had to be fully implemented as of May 2017 [7].

Previous studies found that 28% of dual-users from six EU countries reported having noticed the leaflet [8], while around 16% of dual-users from the United Kingdom noticed the text warning [9]. The current study is the first to examine whether dual-users’ perceptions regarding the addictiveness and toxicity of e-cigarettes changed after implementing the EU’s new legislation.

In sum, this study aims to answer the following research questions: (1) To what extent did Dutch e-cigarette users notice the EU’s new e-cigarette legislation? (2) Did e-cigarette users have different perceptions regarding the addictiveness and toxicity of e-cigarettes after implementing the EU’s new legislation than before its implementation?

## 2. Materials and Methods

### 2.1. Sample

Longitudinal data were used from the International Tobacco Control (ITC) Netherlands Survey Waves 9 (2015), 10 (2016), and 11 (2017), with all online surveys being conducted between November and December. The ITC Netherlands Surveys received ethics clearance from the University of Waterloo’s Office of Research Ethics (ORE # 18920). The ITC Project’s methodology has been described previously [10]. Respondents were selected from a probability-based web database to reach a sample representative of Dutch smokers aged 15 years and older [11]. Tailored replenishment samples and sampling weights were used to compensate for attrition effects [12]. Respondents were classified as smokers if they had smoked at least 100 cigarettes in their lifetime and if they currently smoked at least once a month [13]. Smokers and ex-smokers who had ever heard of e-cigarettes were included. Respondents who reported using e-cigarettes at least monthly were categorized as “e-cigarette users”. The control group included tobacco-only smokers. The number of included respondents was *n* = 1146 in 2015 (of which *n* = 108 e-cigarette users), *n* = 1151 in 2016 (of which *n* = 123 e-cigarette users), and *n* = 1124 in 2017 (of which *n* = 130 e-cigarette users). Five-hundred and thirty-six (of which *n* = 58 e-cigarette users) respondents participated in all three Survey Waves.

### 2.2. Measures

#### 2.2.1. Noticing

From 2016 onward, respondents were asked, “In the last 30 days, have you noticed any health warnings on packaging for e-cigarettes, cartridges, or e-liquid bottles or containers?” The response options were “yes” (coded as 1), “no”, and “don’t know’ (both coded as 0).

Respondents also received the question, “As far as you know, is there health and product safety information contained on leaflets inside the packaging of disposable e-cigarettes, cartridges, or e-liquid?” Again, the response options were “yes” (coded as 1), “no”, and “don’t know” (both coded as 0).

#### 2.2.2. Perceptions

In all Survey Waves, respondents were asked, “Do you think that e-cigarettes are addictive?” and “Do you think that e-cigarettes are toxic?” Response options were (1) “not at all”, (2) “slightly”, (3) “moderately”, (4) “very much”, (5) “extremely”, and “don’t know” (coded as missing).

### 2.3. Statistical Analysis

SPSS 24.0 was used to analyze the data. All statistical estimates and tests were weighted for gender and age to increase sample representativeness [10]. To examine to what extent Dutch e-cigarette users noticed the EU’s new legislation, descriptive statistics were used. Generalized Estimating Equations (GEE) [14] were performed to estimate whether e-cigarette users had different perceptions regarding the addictiveness and toxicity after implementing the new legislation than before its implementation. Tobacco-only smokers were used as a control group for the e-cigarette users by adding interactions between Survey Wave and e-cigarette status; e-cigarette users vs. tobacco-only smoker. The control group is not expected to be exposed to the new e-cigarette legislation. For the GEE analyses, only respondents who participated in all three Survey Waves were included (*n* = 536). The binominal distribution and the logit link were used for the dichotomous variables, while the normal distribution and the identity link were used for the continuous variables [15]. The GEE were adjusted for age, gender, educational level, level of nicotine addiction [16], ever having made a quit attempt, quit intention, the number of participations in the cohort [17], and e-cigarette status (except when interactions were applied).

## 3. Results

### 3.1. Noticing

GEE analyses revealed that, compared to tobacco-only smokers, a higher proportion of e-cigarette users noticed the text warning (OR = 4.006, *p* < 0.001) and knew about the leaflet (OR = 5.530, *p* < 0.001) (not in table). The ORs result from comparing 5.5% (2016) and 4.4% (2017) (tobacco-only smokers) vs. 28.4% (2016) and 26.1% (2017) (e-cigarette users) for noticing the text warning, and from comparing 4.9% (2016) and 6.4% (2017) (tobacco-only smokers) vs. 32.4% (2016) and 33.4% (2017) (e-cigarette users) for knowing about the leaflet (Table 1).

### 3.2. Perceptions

The GEE from Table 1 reveals that respondents reported higher scores on perceptions regarding the addictiveness and toxicity of e-cigarettes after implementing the new legislation than before (Table 1). Significant interactions were found between survey wave and e-cigarette status for addictiveness (*p* < 0.001) and toxicity (*p* = 0.001) (not in table). E-cigarette users showed a greater increase in scores on the perception regarding the addictiveness of e-cigarettes (β = 0.457, *p* = 0.045) than tobacco-only smokers (β = 0.135, *p* < 0.001). Also, e-cigarette users showed no change in scores on the perception regarding the toxicity of e-cigarettes (β = 0.246, *p* = 0.055), while tobacco-only smokers showed a small significant increase (β = 0.071, *p* = 0.010) (Table 1).

## 4. Discussion

To our knowledge, our study was the first to examine if implementing the EU’s new e-cigarette legislation was associated with changes in perceptions regarding the addictiveness and toxicity of e-cigarettes.

Regarding the addictiveness and toxicity of e-cigarettes, we found that e-cigarette users showed somewhat larger increases in these perceptions, and they were more likely to have noticed the new legislation than tobacco-only smokers. This might indicate that the e-cigarette users’ changes in perceptions were due to the new legislation and not due to other public health actions or media attention. The increase in perceptions among tobacco-only smokers may be due to their having an interest in the coverage of e-cigarettes in the media. Nonetheless, these increases were smaller than those among e-cigarette users. However, even e-cigarette users barely noticed the new legislation, as found previously [8,9]. Possibly the leaflet is not optimally placed for exposure (Figure 2). Also, e-cigarette users may not have noticed the text warning, as the amount of text might suggest its being informative about something else instead of a health warning. Previous research has shown that text warnings on tobacco products (with shorter texts) are generally more often noticed by smokers [18] than the text warning on e-cigarette packets (our study).

This study has several limitations. First, although we used longitudinal data, this research was not experimental and, therefore, no firm conclusions can be drawn. Second, our study was exploratory, as our sample consisted of only a small number of e-cigarette users and, therefore, we had insufficient statistical power for some of the analyses. Third, our sample consisted of e-cigarette users who were either current smokers or ex-smokers, and it was therefore not representative of the Dutch population of e-cigarette users. We were unable to examine e-cigarette users who never smoked, as the ITC Netherlands Survey selects a sample representative of Dutch smokers aged 15 years and older. Last, it is uncertain if the time between full implementation and data collection (six months) was long enough for the sample to be exposed to the new legislation.

There is one main implication based on the current study’s results. Although perceptions regarding the addictiveness and toxicity were somewhat stronger among e-cigarette users after implementing the new legislation than before, more research should be conducted on effective communication on and in the packets of e-cigarettes, as e-cigarette users barely noticed the legislation.

## 5. Conclusions

The new e-cigarette text warning and leaflet may not be effective tools to inform e-cigarette users about the addictiveness and toxicity of e-cigarettes. Therefore, future research should examine how to make the EU’s new legislation for e-cigarettes more effective.

## Figures and Tables

**Table 1:** Scores on noticing the text warning and the leaflet in 2016 and 2017, and scores on perceptions of the addictiveness and toxicity of e-cigarettes between 2015 and 2017 with betas of trends resulting from GEE (Generalized Estimating Equations), including Confidence Intervals (CIs). *.

Measure	Total Group	Tobacco-Only Smokers	E-Cigarette Users
Text warning	*n* = 1631	*n* = 1487	*n* = 144
2016 (%)	8.0	5.5	28.4
2017 (%)	6.9	4.4	26.1
Leaflet	*n* = 1631	*n* = 1487	*n* = 144
2016 (%)	7.9	4.9	32.4
2017 (%)	9.5	6.4	33.4
Addictiveness	*n* = 1353	*n* = 1201	*n* = 152
2015 (mean, SD)	2.70 (0.98)	2.76 (1.0.99)	2.24 (0.72)
2016 (mean, SD)	2.75 (0.95)	2.79 (0.95)	2.52 (0.92)
2017 (mean, SD)	2.88 (0.95)	2.90 (0.94)	2.75 (0.98)
β (95% CI)	0.141 (0.088 to 0.194)	0.135 (0.075 to 0.294)	0.457 (0.010 to 0.904)
*p*-value	<0.001	<0.001	0.045
Toxicity	*n* = 1340	*n* = 1195	*n* = 145
2015 (mean, SD)	2.49 (0.99)	2.57 (1.00)	1.88 (0.65)
2016 (mean, SD)	2.44 (0.95)	2.50 (0.93)	2.05 (0.96)
2017 (mean, SD)	2.48 (0.97)	2.54 (0.94)	2.10 (0.87)
β (95% CI)	0.069 (0.018 to 0.120)	0.071 (0.017 to 0.125)	0.246 (−0.005 to 0.498)
*p*-value	0.008	0.010	0.055

* The *n* resulted from the number of observations from the GEE. Data were weighted for gender and age, and all GEE analyses were adjusted for age, gender, educational level, HSI, ever having made a quit attempt, quit intention, the number of times a respondent participated in the cohort, and e-cigarette status (e-cigarette users vs. tobacco smoker; only for the total group, thus not for the stratified analyses).
